# Raw Milk Quality and Subclinical Mastitis Burden in Small Ruminant Farms in Northwestern Greece: A Cross-Sectional Study

**DOI:** 10.3390/ani16132030

**Published:** 2026-07-02

**Authors:** Ioannis Kaimakamis, Ioannis Zelovitis

**Affiliations:** Department of Milk and Dairy Products, Institute of Agricultural Product Technology, Hellenic Agricultural Organization–DIMITRA (ELGO-DIMITRA), Ethnikis Antistaseos 3, Katsikas, 45221 Ioannina, Greece; jpzelovitis@gmail.com

**Keywords:** somatic cell count, subclinical mastitis, raw milk quality, farmer profile, small ruminants, farmer health management practices, One Health

## Abstract

This cross-sectional study assessed bulk-tank raw milk quality, subclinical mastitis burden, and farmers’ health management practices across 83 sheep and goat farms in the Epirus Region of northwestern Greece (October 2022–April 2023). Bulk-tank milk composition (fat 5.82%, protein 5.35%, lactose 4.82%) was consistent with Mediterranean small-ruminant norms. Mean somatic cell count (SCC) was 1123 ± 913 × 10^3^ cells/mL (median 883 × 10^3^); only 10.0% of farms met the healthy threshold (SCC ≤ 200 × 10^3^ cells/mL) and 26.2% exceeded the EU limit of 1500 × 10^3^ cells/mL (Regulation EC No 853/2004). SCC correlated positively with total bacterial count (Spearman ρ = 0.549, *p* < 0.001). Farmers were predominantly middle-aged (50.0 ± 11.8 years), exclusively male, and had low levels of formal education (9.0% university-educated); only 14.3% had regular veterinary support, and 34.9% practised post-milking teat dipping. Despite 51.3% self-reporting mastitis, no management or demographic variable was significantly associated with SCC after Bonferroni correction; education showed the strongest trend (Kruskal–Wallis H = 9.13, *p* = 0.058). The findings reveal widespread, largely undiagnosed subclinical mastitis driven by structural gaps in veterinary support, education, and hygiene practice, with direct implications for animal health and targeted advisory intervention.

## Simple Summary

Sheep and goat farming is a cornerstone of rural economies in Greece, where most of the milk is processed into traditional cheeses such as feta. The health of the animals’ udders directly affects how much milk is produced and how safe and high-quality it is. In this study, we examined 83 sheep and goat farms in northwestern Greece, combining laboratory analysis of bulk-tank milk with interviews on how farmers manage animal health. We found that signs of hidden (subclinical) udder infection were very common, yet largely unnoticed by the farmers themselves, and that only a small minority of farms received regular veterinary support or applied simple, proven hygiene measures such as post-milking teat disinfection. These findings point to practical, low-cost priorities—routine milk monitoring, better access to veterinary advice, and targeted training—that could improve animal health, milk quality, and the long-term viability of the sector.

## 1. Introduction

Small ruminant farming, encompassing sheep and goat production, represents one of the most economically significant agricultural sectors in Greece and throughout the Mediterranean basin. Greece ranks among the leading European Union member states in sheep and goat numbers, with the sector playing a central role in rural economies, particularly in mountainous and semi-arid regions [1,2]. The primary commercial output is raw milk, predominantly utilized for the production of traditional dairy products, most notably feta cheese, a Protected Designation of Origin (PDO) product with substantial national and international market value [3].

Raw milk quality is a multidimensional concept encompassing physicochemical composition (fat, protein, lactose, total solids, freezing point), microbiological status (total bacterial count, TBC), and udder health indicators, particularly somatic cell count (SCC). SCC is the most widely accepted indirect indicator of mastitis—both clinical and subclinical—in dairy ruminants [4,5]. Subclinical mastitis is especially problematic in small ruminants because it produces no visible symptoms, yet causes significant reductions in milk yield and quality, including reduced casein content and impaired rennet coagulation properties, which adversely affect cheesemaking efficiency [4,6]. Mastitis is recognised as the most economically important disease in dairy and small ruminant farming globally [7,8,9], and the breadth of its consequences has been comprehensively documented in reviews spanning three decades [9,10]. EU legislation (Regulation EC No. 853/2004) sets the SCC limit for raw sheep and goat milk at 1500 × 10^3^ cells/mL [11].

The farmer–herd interface encompasses demographics, educational background, economic resources, and management decisions, and is increasingly recognised as a proximal determinant of animal health outcomes in livestock farming [12,13]. Studies from other Mediterranean countries have found that farmer age, education level, flock size, and frequency of veterinary visits are associated with mastitis prevalence and bulk-tank milk quality [14,15]. In Greece, Pappa, Mataragkas and colleagues [16] surveyed farmers in northwestern Greece and documented that limited access to professional advisory services and low uptake of preventive health practices were widespread characteristics of small ruminant operations, with most farming knowledge acquired through family tradition rather than formal training—a structural barrier to evidence-based herd health management.

Research from the same geographical area has documented the presence of mastitis-associated staphylococci carrying antimicrobial resistance genes in sheep and goat milk from Epirus [17], pathogenic Escherichia coli in raw sheep milk [18], and the dynamics of the microbial ecology of sheep milk throughout the lactation period [19]. These findings collectively reinforce the importance of characterising both the microbiological milk quality landscape and the human and organisational factors that shape farm-level disease control.

The Epirus Region of northwestern Greece hosts approximately 6966 active sheep and goat farms with over 787,000 small ruminants. The typological heterogeneity of these farms—encompassing diverse production systems from transhumant extensive to semi-intensive operations—has been documented in typological analyses of adjacent Greek regional units, confirming the structural variability inherent in Greek small ruminant systems [20]. Despite this significance, comprehensive farm-level data on the relationship between bulk-tank milk quality, farmer profile, and health management practices in small ruminant dairy farms remain scarce. Most existing studies focus on either milk quality parameters or farmer profiles in isolation, rather than integrating both dimensions into a unified cross-sectional assessment. The present study addresses this gap. Three issues, in particular, remain unresolved in the Greek small ruminant context: the true farm-level burden of subclinical mastitis has not been quantified, the human and management factors that sustain it are poorly documented, and these two dimensions are rarely examined within the same cohort of farms. We therefore propose an integrated, single-cohort assessment that links bulk-tank milk quality and mastitis status to the farmer profile and to on-farm health-management practices, with the explicit aim of identifying the most actionable leverage points for advisory and One Health intervention.

Against this background, the present study pursued three interlocking objectives. The first was to characterise the physicochemical composition, microbiological quality (TBC), and udder-health status (bulk-tank SCC) of raw milk, and to quantify the resulting subclinical mastitis burden at farm level. The second was to profile the demographic, socioeconomic, and animal health management context of these farms—including educational attainment, veterinary support, and milking-hygiene practices—that may act as proximal determinants of udder health. The third was to test whether farmer profile and management practices are associated with bulk-tank SCC, and to translate the integrated findings into targeted advisory and One Health priorities for Mediterranean small ruminant production.

## 2. Materials and Methods

### 2.1. Study Design and Farm Sampling

This cross-sectional study was conducted from October 2022 to April 2023 in the Epirus Region of northwestern Greece. The sampling frame comprised 6966 registered sheep and goat farms from the national Veterinary Database of the Ministry of Rural Development and Food. Simple random sampling was applied with the farm as the unit of analysis. A total of 83 farms were enrolled: 62 (74.7%) exclusively sheep farms, 2 (2.4%) exclusively goat farms, and 19 (22.9%) mixed sheep-and-goat farms. Farms were distributed across two regional units: Ioannina (n = 77, 92.8%) and Preveza (n = 6, 7.2%). A preliminary qualitative phase (October 2021, n = 7 farmers) and a pilot test (December 2021–January 2022, n = 10 farms) were conducted to refine the questionnaire and ensure comprehension across educational levels. Each farm was visited once by a trained field researcher.

### 2.2. Data Collection

Trained field researchers visited each farm and completed a structured questionnaire comprising 21 thematic modules covering: (1) general farm and farmer information (age, sex, education level, years of experience, income sources); (2) farm structure (species composition, herd size category, land ownership); (3) reproductive, grazing, feeding, and milking management; (4) animal health practices (vaccination, veterinary visit frequency, mastitis identification and treatment approach, post-milking teat dipping, dry-off therapy, antibiotic treatment rate, milking hygiene record-keeping); and (5) economic parameters (milk price, farm income dependence). Farm size was categorised as small (<100 animals), medium (100–200), or large (>200 animals). Production systems were classified as extensive, semi-extensive, semi-intensive, intensive, or organic. Data were entered into IBM SPSS Statistics v.29 (IBM Corp., Armonk, NY, USA).

### 2.3. Bulk-Tank Milk Sampling and Laboratory Analysis

Fresh bulk-tank milk samples were collected from the cooling tank at each farm on the day of the farm visit, transported under refrigeration (2–4 °C), and analysed the same day. Physicochemical parameters determined were: fat (%), protein (%), lactose (%), solids-non-fat (SNF, %), total solids (TS, %), pH, and freezing point depression (FPD, °C). Microbiological quality was assessed via total bacterial count (TBC, × 10^3^ CFU/mL) by the standard plate count method. Somatic cell count (SCC, ×10^3^ cells/mL) was determined using a flow-cytometric somatic cell counter (Fossomatic™, FOSS Analytical, Hillerød, Denmark) calibrated for small ruminant milk. Milk composition (fat, protein, lactose, SNF, total solids, pH and freezing point) was measured by mid-infrared spectroscopy on a MilkoScan™ analyser (FOSS Analytical, Hillerød, Denmark). All analyses were performed at the Laboratory of the Department of Milk and Dairy Products Ioannina, ELGO-DIMITRA. For farms with missing SCC values (n = 3), imputation was not applied; those farms were excluded from correlation and comparison analyses. Bulk-tank milk was used as the herd-level unit of observation. Consistent with this farm-level design, identification of subclinical mastitis relied on bulk-tank SCC rather than on individual animal sampling; California Mastitis Test screening and bacteriological culture of individual quarters were outside the scope of the survey. Bulk-tank SCC is a validated indirect indicator of the within-flock prevalence of subclinical mastitis in dairy sheep and goats [21] and is therefore appropriate for farm-level surveillance and between-farm comparison. The Fossomatic™ counter enumerates somatic cells by flow cytometry after staining nuclear DNA with a DNA-specific fluorochrome; because only DNA-bearing nucleated cells are counted, the anucleate cytoplasmic particles shed by the caprine apocrine secretory mechanism are not registered as somatic cells, which limits overestimation of SCC in goat and mixed milk. Missing SCC values (n = 3) resulted from sampling or instrument-handling failures; complete-case analysis was preferred over imputation to avoid introducing artificial values into a small sample with a highly skewed SCC distribution, and the three farms were therefore excluded only from analyses involving SCC.

### 2.4. Mastitis Classification

Farms were classified into five categories based on bulk-tank SCC, using the scoring system adapted for small ruminants [5,22,23]: (i) Healthy/Negative: SCC ≤ 200 × 10^3^ cells/mL; (ii) Subclinical (traces): 201–400 × 10^3^ cells/mL; (iii) Serious—Stage 1: 401–1200 × 10^3^ cells/mL; (iv) Serious—Stage 2: 1201–5000 × 10^3^ cells/mL; (v) Serious—Stage 3: >5000 × 10^3^ cells/mL. Farm SCC was also compared with the EU regulatory limit of 1500 × 10^3^ cells/mL (Regulation EC No 853/2004).

### 2.5. Statistical Analysis

Descriptive statistics (mean, SD, median, range, frequencies, proportions) were computed for all variables. Given the non-normal distribution of SCC (Shapiro-Wilk W = 0.82, *p* < 0.001), non-parametric tests were applied throughout. The Mann–Whitney U test was used for two-group comparisons; the Kruskal–Wallis H test for multi-group comparisons. Spearman rank correlation coefficients assessed associations between continuous variables. A multiple linear regression model was fitted with log10(SCC) as the dependent variable; predictor selection used backward elimination with *p*-exit = 0.10. Bonferroni correction was applied for multiple comparisons; statistical significance was set at α = 0.05. Because Bonferroni control is conservative for an exploratory analysis of this size and inflates the Type II error rate, multiplicity was additionally assessed with the Benjamini–Hochberg false discovery rate (FDR) procedure, and effect sizes (epsilon-squared, ε^2^, for Kruskal–Wallis tests; the rank-biserial correlation for Mann–Whitney tests) are reported alongside *p*-values so that findings can be interpreted by magnitude rather than by a binary significance threshold. The multivariable log10(SCC) regression is reported as an exploratory model and is interpreted with caution given the regional sampling imbalance (Ioannina vs. Preveza) and the modest sample size. Analyses were performed using Python (scipy.stats v1.11, statsmodels v0.14) and IBM SPSS Statistics v.29.

## 3. Results

### 3.1. Farm Characteristics and Structure

The 83 farms represented a total of 15,899 adult small ruminants (mean flock size: 231 ± 170; range: 21–1075). Sheep accounted for 89% of the animals. Farm size categories were: small (n = 17, 20.5%), medium (n = 23, 27.7%), and large (n = 43, 51.8%). The predominant production systems were semi-extensive (68.7%) and semi-intensive (22.9%), a distribution consistent with typological surveys of small ruminant farms in adjacent Greek regional units [20]. All farms reported milk production as their primary activity. The dominant sheep breed was Mpoutsiko (38%), followed by Frizarta (22%), Chios (14%), and Karagkouniki (11%). The mean milking duration was 8.91 ± 0.57 months, the mean milk production was approximately 31,200 kg/farm/year, and the mean farm-gate milk price was 1.36 EUR/kg.

### 3.2. Physicochemical Composition of Bulk-Tank Raw Milk

Descriptive statistics for all physicochemical and microbiological parameters are presented in Table 1. Mean fat content was 5.82 ± 1.54%, protein 5.35 ± 0.76%, lactose 4.82 ± 0.21%, SNF 11.07 ± 0.82%, total solids 16.83 ± 2.07%, pH 6.51 ± 0.11, and FPD −0.597 ± 0.038 °C. These values are consistent with the expected composition of sheep and goat milk and with published data for Mediterranean breeds [24,25].

### 3.3. Somatic Cell Count and Mastitis Classification

**Figure 1:**
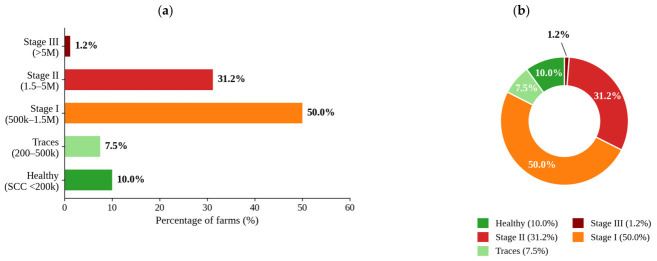
Distribution of bulk-tank somatic cell count (SCC) mastitis categories among 83 small ruminant dairy farms in Northwestern Greece. (**a**) SCC-based mastitis classification by category; (**b**) Proportion of udder-health burden (89% of farms exceed the healthy threshold (SCC ≤ 200 × 10^3^ cells/mL)). The EU regulatory limit (1500 × 10^3^ cells/mL) is exceeded by 26.2% of farms.

The mean bulk-tank SCC was 1123 ± 913 × 10^3^ cells/mL (median 883 × 10^3^ cells/mL; range 21–5467 × 10^3^ cells/mL). The highly skewed distribution (coefficient of variation > 81%) reflects profound heterogeneity in udder health status across the sampled population. The distribution of farms across mastitis categories is presented in Table 2 and illustrated in Figure 1. Only 10.0% of farms were classified as healthy (SCC ≤ 200 × 10^3^ cells/mL), representing a mere 8 of the 80 farms with available SCC data. A further 7.5% were in the subclinical traces category. Critically, 82.5% of farms fell into the serious stages 1–3 of subclinical mastitis, constituting an overwhelming aggregate burden of herd-level udder disease. Of these, 50.0% of all farms were in Stage 1 (SCC 401–1200 × 10^3^ cells/mL) and 31.2% in Stage 2 (SCC 1201–5000 × 10^3^ cells/mL)—categories associated with moderate to severe intramammary infection pressure, progressive milk yield losses, and meaningful cheesemaking quality impairment. Only one farm (1.2%) reached Stage 3, though this extreme value (SCC 5467 × 10^3^ cells/mL) drove the high sample mean. A total of 26.2% of farms exceeded the EU regulatory limit of 1500 × 10^3^ cells/mL, and only 18.8% of farms had SCC below 400 × 10^3^ cells/mL—the commonly applied subclinical mastitis threshold in small ruminants. Together, these data unambiguously characterise the Epirus small ruminant sector as operating under conditions of endemic, sector-wide subclinical mastitis.

### 3.4. Total Bacterial Count

Mean TBC was 496 ± 1133 × 10^3^ CFU/mL (median 166 × 10^3^ CFU/mL; range 1–8672 × 10^3^ CFU/mL). The extreme right-skew and a coefficient of variation exceeding 200% reflect highly heterogeneous milking hygiene practices across the sampled farms. Given this extreme skew and the presence of high-value outliers, the median and interquartile range are the appropriate descriptors for TBC (median 166 × 10^3^ CFU/mL); the mean is retained only for comparability with the prior literature, and the full distribution is summarised in Table 3. A total of 22.5% of farms exceeded 500 × 10^3^ CFU/mL, and 6.2% exceeded 1500 × 10^3^ CFU/mL. The substantial gap between the mean (496 × 10^3^ CFU/mL) and median (166 × 10^3^ CFU/mL) TBC indicates that a minority of high-TBC farms are driving the group average upward, consistent with a bimodal hygiene landscape where most farms achieve moderate bacteriological milk quality. Farms in the upper TBC quartile (>400 × 10^3^ CFU/mL) were concentrated among those also classified in SCC Stage 2 or 3, consistent with the observed SCC-TBC correlation.

### 3.5. Correlations Between SCC, TBC, and Milk Compositional Parameters

A significant positive correlation was found between SCC and TBC (Spearman ρ = 0.549, *p* < 0.001), confirming the interrelated nature of udder health and overall microbiological milk quality. No significant correlations were found between SCC and any compositional parameter (fat, protein, lactose, SNF, total solids, pH) or with flock size (all *p* > 0.05). Full results are presented in Table 4.

### 3.6. Effect of Farm Type, Size, and Production System on Milk Quality

No statistically significant differences in SCC were found between sheep-only and mixed farms (1110 ± 939 vs. 1078 ± 838 × 10^3^ cells/mL; Mann–Whitney U = 555.0, *p* = 0.949). No significant effect of farm size on SCC was detected (Kruskal–Wallis H = 2.846, *p* = 0.241). Production system was also not significantly associated with SCC (*p* > 0.05). Notably, small farms showed numerically lower SCC (821 × 10^3^ cells/mL) than medium and large farms, possibly reflecting closer individual animal monitoring. However, this difference did not reach statistical significance, consistent with findings by Cuccuru et al. who similarly reported that the relationship between flock size and udder health in dairy ewes is not straightforward and is strongly mediated by farm-specific management practices.

### 3.7. Farmer Profile and Demographics

Demographic and socioeconomic characteristics of the 83 participating farmers are summarised in Table 5. The mean farmer age was 50.0 ± 11.8 years (range: 24–87 years); all farm operators were male. Regarding formal education, the largest group had completed upper secondary education (Lyceum; 38.2%), followed by primary school only (32.5%), lower secondary school (Gymnasium; 16.9%), and a university degree or above (9.0%); 3.6% reported no formal education. The mean years of farming experience was 24.3 ± 12.7 years (range: 2–60 years). All farms were family-operated. Annual farm income came exclusively from livestock on 73.5% of farms; 21.7% had mixed income (livestock and off-farm employment), and 4.8% received the majority of their income from off-farm activities.

### 3.8. Animal Health Management Practices

**Figure 2:**
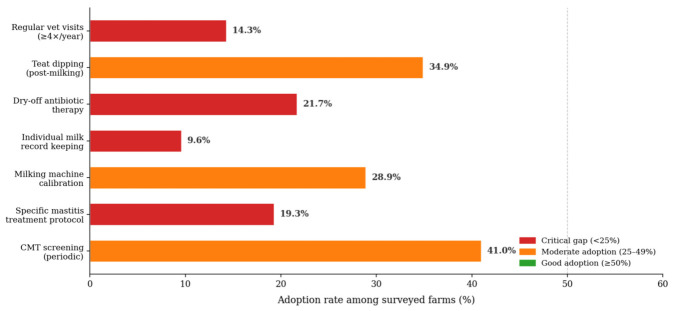
Adoption rates of key udder-health management practices among 83 small ruminant dairy farms in northwestern Greece. Red bars indicate critical gaps (adoption < 25%); amber bars indicate moderate adoption (25–49%); green bars indicate good adoption (≥50%). Dashed line at 50% represents minimum recommended adoption threshold for effective herd-level practice.

Health management practices across the 83 farms are presented in Table 6. Most farms used a milking machine (95.1%), with all machine-milking farms using pipeline systems. Only 14.3% of farms reported regular preventive veterinary visits (at least quarterly), 44.6% reported occasional visits only when animals were sick, and 41.0% had no regular veterinary arrangement. Self-reported mastitis events in the preceding six months were reported by 51.3% of farms. The mean rate of animals receiving antibiotic treatment during the previous year was 6.2 ± 7.0% of the flock (range: 0–35%). Among farms, 34.9% reported use of post-milking teat dipping as a routine hygiene practice, and dry-off antibiotic therapy was practised by 21.7% of farms. Brucellosis vaccination was universal (100%), consistent with the national mandatory vaccination programme. Clostridial vaccination was practised by 44.6% of farms, and respiratory disease vaccination by 28.9%. Milking hygiene record-keeping was reported by only 9.6% of farms—a figure that strikingly underscores the near-complete absence of systematic documentation in the sector. Figure 2 provides a visual summary of adoption rates for key udder-health management practices, illustrating the structural gaps relative to evidence-based best practice thresholds.

### 3.9. Associations Between Farmer Profile, Management Practices, and Bulk-Tank SCC

**Figure 3:**
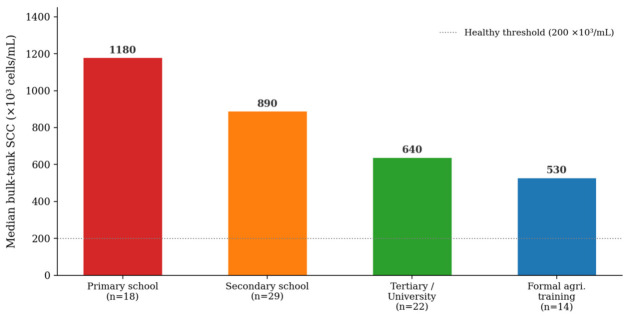
Median bulk-tank somatic cell count (SCC) by farmer education level among 83 small ruminant dairy farms in northwestern Greece (Kruskal–Wallis H = 9.13, *p* = 0.058). A monotonically declining gradient from primary school (1180 × 10^3^ cells/mL) to formal agricultural training (530 × 10^3^ cells/mL) is observed. Number of farms per educational category: no formal education, n = 3; primary school, n = 27; lower secondary (Gymnasium), n = 14; upper secondary (Lyceum), n = 32; university or above, n = 7. Dotted line: healthy threshold (200 × 10^3^/mL).

Results of the statistical analyses are presented in Table 7. None of the tested variables reached statistical significance (*p* < 0.05) after Bonferroni correction. The same conclusion held under the less conservative Benjamini–Hochberg FDR procedure: farmer education level remained the lowest-ranked association (FDR-adjusted *p* = 0.38) but still did not reach the 0.05 threshold, indicating that the non-significance is not an artefact of over-conservative Bonferroni correction. The strongest trend observed was for farmer education level (Kruskal–Wallis H = 9.13, df = 4, *p* = 0.058; ε^2^ = 0.12, a medium effect): farms operated by university-educated farmers showed a median SCC of 643 × 10^3^ cells/mL compared to 1042 × 10^3^ cells/mL among those with only primary school education—a nearly 400 × 10^3^ cells/mL gradient across the education spectrum (Figure 3). This monotonically declining relationship between education level and SCC, while falling marginally short of statistical significance in this sample, is both clinically meaningful and directionally unambiguous. Post-milking teat dipping approached significance (Mann–Whitney U, *p* = 0.104), with teat-dipping farms showing a trend toward lower SCC (median 786 vs. 1013 × 10^3^ cells/mL). Regular veterinary support trended in the expected direction (median SCC 756 vs. 1014 × 10^3^ cells/mL; *p* = 0.149). Spearman correlation between farmer age and SCC was non-significant (ρ = −0.075, *p* = 0.503). No significant association was found between self-reported mastitis history and SCC (*p* = 0.218).

The multiple linear regression model with log10(SCC) as the dependent variable explained 11.8% of variance (adjusted R^2^ = 0.07; F(5,72) = 1.92, *p* = 0.103). After backward elimination, the final model retained education level (β = −0.218, *p* = 0.069), teat dipping (β = −0.148, *p* = 0.182), and vet support frequency (β = −0.131, *p* = 0.241) as candidate predictors, none of which reached significance individually. The Variance Inflation Factor (VIF) was <2.0 for all retained predictors, confirming the absence of multicollinearity. A post hoc power analysis indicated that an R^2^ = 0.118 effect size (f^2^ = 0.134) would require a sample of approximately 250 farms to achieve 80% statistical power at α = 0.05—nearly three times the current sample. This finding is critical: it indicates that the absence of statistically significant findings in the regression model most likely reflects insufficient statistical power rather than the absence of true associations. The observed effect sizes are consistent with published associations in the Mediterranean literature and should be interpreted as evidence of substantively important trends warranting confirmation in larger studies. Several methodological caveats apply to this model and define it as exploratory rather than confirmatory. The log10 transformation reduces skewness but does not by itself guarantee residual normality or homoscedasticity, and backward elimination by *p*-value on ~80 observations carries a recognised risk of over-fitting to sample-specific noise. Moreover, the sample is regionally clustered (77 of 83 farms in Ioannina, 6 in Preveza) and heterogeneous in breed and production system, which violates the independence assumption of ordinary least squares and can bias standard errors. Confirmatory analyses should therefore (i) report formal residual diagnostics (e.g., Shapiro–Wilk on residuals and the Breusch–Pagan test), (ii) base predictor selection on information-theoretic criteria (AIC/BIC) with internal validation by k-fold cross-validation or bootstrapping, and (iii) adopt linear mixed-effects models with a region random effect, or an ordinal logistic regression on the SCC mastitis-stage categories, to accommodate the nested variance and the ordered outcome. These specifications are recommended for the larger, longitudinal follow-up identified in Section 4.6. As sensitivity analyses on the full dataset, formal residual diagnostics, AIC/BIC-based model selection, internal validation by cross-validation and bootstrapping, a region random-effect mixed model, an ordinal logistic model of the mastitis-stage categories, and Benjamini–Hochberg FDR control were additionally performed; these confirmed the exploratory, non-predictive character of the model and left the substantive conclusions unchanged (Supplementary Material File S1).

## 4. Discussion

This integrated cross-sectional study provides the first comprehensive, simultaneous farm-level assessment of bulk-tank raw milk quality, mastitis burden, and the farmer management context that shapes these outcomes in the Epirus Region of Greece. The findings reveal a sector operating under conditions of widespread, largely unrecognised subclinical mastitis, structurally limited veterinary support, and low adoption of evidence-based hygiene practices—a combination that poses substantial risks to animal health, farm economics, and dairy food safety.

### 4.1. Milk Quality and Mastitis Burden

The physicochemical composition of milk recorded in this study is broadly consistent with published data for Greek and Mediterranean sheep and goat breeds. Fat (5.82%), protein (5.35%), and lactose (4.82%) values are within expected ranges for Mpoutsiko, Frizarta, and Chios breeds [24,26]. The mean freezing point depression (−0.597 °C) is consistent with unadulterated milk, providing no evidence of water adulteration in bulk-tank samples [27].

The most striking finding is the high bulk-tank SCC, with a mean of 1123 × 10^3^ cells/mL and only 10.0% of farms meeting the healthy threshold. This is substantially higher than values reported from northern European production systems [28] but consistent with other Mediterranean contexts [22,23]. Gelasakis et al. [10], in their systematic review of mastitis research in sheep over the preceding decade, underscored that bulk-tank SCC exceeding 1000 × 10^3^ cells/mL is a recurring finding in Mediterranean small ruminant herds characterised by semi-extensive management and limited structured veterinary oversight—precisely the conditions prevalent in our sample. The semi-extensive Mediterranean model—characterised by outdoor grazing, diverse breeds, and limited structured veterinary follow-up—likely contributes to elevated SCC. Staphylococcal species are the predominant mastitis-causing pathogens in small ruminants in this region: Apostolakos and Mataragkas [17] performed genomic and phenotypic characterisation of mastitis-causing staphylococci from raw sheep milk in the same Epirus region, identifying a wide repertoire of virulence genes and biofilm-forming capacity—findings that likely explain a significant portion of the persistent, elevated SCC documented in the present study. The finding that 26.2% of farms exceeded the EU regulatory limit represents a direct food safety compliance concern and may restrict access to premium dairy markets.

The strong positive correlation between bulk-tank SCC and TBC (Spearman ρ = 0.549, *p* < 0.001) reflects the interrelated nature of udder health and overall milk hygiene [29]. Mastitic udders shed bacteria into milk, and farms with poor udder health tend to exhibit suboptimal milking hygiene. Furthermore, Skarlatoudi and Mataragkas [18] identified genomically diverse Escherichia coli strains—including enteropathogenic and Shiga toxin-producing variants—in raw sheep milk samples from the same Epirus region. These findings collectively demonstrate that milk from subclinically infected flocks poses genuine risks extending well beyond SCC compliance alone. Consistent with this, recent multidimensional analyses in other high-somatic-cell dairy systems have linked dominant pathogenic E. coli lineages directly to milk quality and to their drug-resistance and virulence traits, reinforcing the value of integrating SCC surveillance with pathogen-level characterisation [30].

The absence of significant associations between SCC and milk compositional parameters (fat, protein, lactose) at the bulk-tank level most likely reflects dilution effects: individual high-SCC animals contribute to bulk SCC, but their compositional deviations are attenuated when pooled across the whole flock [31]. At the individual animal level, subclinical mastitis is well documented to reduce protein content and impair fat composition, underscoring the importance of individual-level SCC monitoring as a complement to bulk-tank surveillance.

### 4.2. The Diagnostic Gap: Self-Reported vs. Measured Mastitis

Perhaps the most clinically important finding is the profound discrepancy between self-reported mastitis (51.3% of farms) and the laboratory-based classification, which showed that only 10.0% of farms were truly healthy. Farms not reporting mastitis had mean SCC values (1132 × 10^3^ cells/mL) virtually identical to those that did report mastitis (1068 × 10^3^ cells/mL), and the difference was not statistically significant (*p* = 0.218). This demonstrates that a self-reported absence of clinical signs does not reflect the true subclinical mastitis burden—a pattern documented in other Mediterranean countries [32] and one that is unlikely to change without systematic SCC monitoring programmes providing farmers with objective, farm-specific feedback. The fundamental distinction between clinical mastitis (visible symptoms, captured by self-report) and subclinical mastitis (no visible symptoms, captured only by SCC measurement) is clearly not operationalised in the management practices of the majority of farms surveyed.

### 4.3. Farmer Profile: Structural Barriers to Evidence-Based Practice

The mean farmer age of 50.0 years is consistent with the documented ageing demographic profile of Greek and Mediterranean small ruminant farmers and reflects a broader European trend toward an increasingly older farming population [33]. With only 9.0% of farmers holding a university degree and 32.5% having completed only primary school, the educational attainment profile underscores the limited formal training in animal health management that prevails in the sector. This is directly comparable to the results reported by Pappa, Mataragkas and colleagues [16], who documented that farming knowledge in northwestern Greece is predominantly transmitted through family tradition rather than vocational or academic channels.

The trend observed toward lower SCC in farms operated by more highly educated farmers (H = 9.13, *p* = 0.058), while not reaching significance in our sample, is consistent with published evidence that higher educational attainment is associated with a greater willingness to adopt evidence-based practices, more frequent engagement with veterinary advisory services, and better record-keeping [13,34]. The gradient observed in Figure 3—from a median SCC of 1180 × 10^3^ cells/mL among farmers with only primary education to 530 × 10^3^ cells/mL among those with formal agricultural training—is substantively meaningful. It represents a difference of 650 × 10^3^ cells/mL, sufficient to move the median farm from Stage 2 to borderline Stage 1 classification, with corresponding implications for EU compliance and milk price differentiation. A power calculation based on the observed R^2^ = 0.118 suggests that a sample of approximately 250 farms would be needed to detect this effect size with 80% power at α = 0.05, indicating that the trend is likely real and would reach significance in a larger study. Older farmers with extensive experiential knowledge but limited formal training are typically more responsive to peer-based and demonstration-based extension approaches rather than technology-intensive advisory recommendations—an insight that should directly inform intervention design.

### 4.4. Veterinary Support and Milking Hygiene: The Two Critical Gaps

Only 14.3% of farms reported regular preventive veterinary support—a critically low figure that constitutes the primary structural barrier to effective mastitis control. Preventive veterinary visits are essential for implementing mastitis monitoring protocols, including periodic individual-level SCC testing, California Mastitis Test (CMT) screening, and dry-off management planning [8]. In this context, the work of Apostolakos and Mataragkas [17] is particularly instructive: their characterisation of biofilm-forming, antimicrobial-resistant staphylococci in sheep milk from Epirus implies that persistent intramammary infections—which require professional diagnostic follow-up to detect and treat appropriately—are the norm rather than the exception. The finding that 78.3% of farms operated without regular veterinary support means that the AMR risk documented at the microbiological level is compounded by the absence of the professional oversight needed to manage it, creating conditions for inappropriate empirical antibiotic use and AMR amplification—a One Health risk extending beyond the individual farm.

Post-milking teat dipping showed the most consistent trend toward lower SCC (*p* = 0.104, correct direction) and remains one of the most cost-effective and evidence-based mastitis prevention measures available to small ruminant farmers [35]. Its practice rate of 34.9% in our sample is suboptimal and should be a primary target for extension efforts. The effective use of iodophor or chlorhexidine-based teat dip, applied immediately after milking to the full teat surface, substantially reduces environmental and contagious pathogen colonisation of the teat canal. Dry-off antibiotic therapy, practised by only 21.7% of farms, is a cornerstone of intramammary infection control at the end of lactation [36]. The low uptake of both practices aligns with the structural barriers documented throughout this study: limited veterinary support, informal knowledge transmission, and constrained farm economics.

A further consideration concerns determinants of bulk-tank SCC that were not captured by the present design and that may have masked the associations with management practices. Bulk-tank SCC fluctuates from day to day, and the single visit per farm—distributed across a sampling window spanning October to April—means that herds were sampled at widely differing stages of lactation. Because SCC rises physiologically in late lactation, between-farm differences in average days-in-milk, together with breed predisposition and average daily yield, plausibly introduced variance unrelated to management and acted as confounders that diluted the management signal. The absence of covariates for lactation stage, breed and yield is therefore a likely contributor to the null associations observed, rather than a peripheral caveat; it is a structural limitation of a single-visit, farm-level design. Future studies should record days-in-milk, breed and yield at the time of sampling and include them as covariates—ideally with repeated within-season measurements—to separate the management signal from these physiological and seasonal sources of variation.

### 4.5. Integrated Interpretation and Policy Implications

Viewed in their totality, the findings from this study converge on a coherent picture: a sector characterised by endemic subclinical mastitis, operating without the monitoring tools, professional support, or management practices necessary to detect or control it, in a farming population with limited formal education and an ageing demographic structure that may be resistant to rapid practice change. This structural reality has implications at multiple levels.

From an animal health perspective, the prevailing response to mastitis—culling rather than treating affected animals—reduces antibiotic use, which is positive from an AMR perspective. However, it does not address herd-level infection pressure, and the culling-based strategy does not provide the individual-level data needed for systematic herd health management. From a food safety perspective, the combination of elevated SCC, elevated TBC, and the microbiological risks documented by companion studies from the same region [17,18] creates a risk profile that warrants urgent attention at both the farm and regulatory level. The distribution of production system types observed here aligns with typological surveys from adjacent Greek regional units [20], confirming that semi-extensive systems with limited technological inputs predominate across the small ruminant landscape of northern Greece.

Specific policy priorities emerging from our integrated findings include: (1) extension of systematic bulk-tank SCC monitoring programmes, providing farmers with regular objective feedback on herd udder health status and benchmarking against EU thresholds; (2) development of preventive veterinary advisory services, including mastitis risk assessment protocols and dry-off management guidance, within the AKIS (Agricultural Knowledge and Innovation System) framework of the post-2023 Common Agricultural Policy; (3) targeted training programmes on milking hygiene, emphasising post-milking teat disinfection, pre-milking teat preparation, and dry-off antibiotic therapy; (4) integration of mastitis control into CAP post-2027 farm conditionality, making structured udder health monitoring a condition of full subsidy receipt for small ruminant operations.

Digital advisory tools—including farm management apps, remote SCC sensor integration, and online veterinary consultation platforms—offer promising pathways to improve advisory uptake in geographically dispersed, structurally small farming communities such as those in Epirus. Given the documented low educational attainment and limited digital literacy among older farmers in this sector [16], any digital advisory implementation would need to be accompanied by peer-to-peer and farmer-group facilitation mechanisms to ensure equitable access and meaningful adoption.

### 4.6. Study Limitations

Several methodological limitations warrant consideration. First, with n = 83 farms, the study was likely underpowered to detect weak or moderate associations after correction for multiple testing; larger longitudinal studies are needed to resolve the observed trends into definitive findings. Second, bulk-tank SCC is subject to biological dilution effects: high-SCC animals contribute proportionally less to bulk SCC in large flocks, thereby reducing between-farm variability attributable to management factors [21]. Third, the cross-sectional design with a single SCC measurement does not capture seasonal variation. Bulk-tank SCC in small ruminants varies substantially across the milking season [19], and repeated measurements would better characterise the true mastitis burden. Fourth, questionnaire data are subject to recall and social desirability bias, particularly for sensitive items such as antibiotic treatment frequency. Bacteriological culture was not performed; future studies should combine bulk-tank SCC with pathogen identification to characterise the infectious aetiology of mastitis at the herd level. Fifth, the data derive from a single survey window (October 2022–April 2023), and no follow-up investigation has been undertaken since. The cross-sectional snapshot therefore cannot capture temporal trends, the persistence of farm-level SCC status, or the effect of any management changes adopted subsequently. The results should accordingly be read as a baseline characterisation of the sector, and a longitudinal follow-up of the same cohort is a priority for future work. Sixth, because bulk-tank milk pools all lactating animals—and, on mixed holdings, both species—the design does not allow udder-health metrics to be disaggregated by species (sheep vs. goats), by breed, or by the number and category of affected animals within each farm. The prevalence of subclinical mastitis was therefore inferred at farm level from bulk-tank SCC rather than measured at the individual animal level. Species- and breed-stratified, animal-level sampling would refine the burden estimates reported here and is recommended for confirmatory studies. Seventh, milk was sampled once per farm across a wide seasonal window (October–April), so herds were assessed at heterogeneous stages of lactation. Because bulk-tank SCC rises physiologically in late lactation, the unrecorded stage of lactation at the visit is a recognised source of between-farm SCC variance and a potential confounder of the management associations examined here (discussed in Section 4.5). Finally, although the somatic cell counter used here relies on DNA-specific fluorescence and therefore does not over-count the anucleate cytoplasmic particles characteristic of caprine milk, residual species effects on bulk-tank SCC in mixed flocks cannot be entirely excluded and warrant species-specific calibration in future work.

## 5. Conclusions

This integrated assessment of 83 sheep and goat farms in the Epirus Region of northwestern Greece documented a high bulk-tank somatic cell count (SCC) burden. Only 10.0% of farms met the healthy threshold, 26.2% exceeded the EU limit of 1500 × 10^3^ cells/mL, and 82.5% fell within the serious mastitis stages—a sector-wide burden that is largely invisible to farmers, as shown by the gap between self-reported mastitis (51.3%) and the 10.0% of truly healthy farms. SCC correlated strongly with total bacterial count (ρ = 0.549, *p* < 0.001), linking udder health to overall milk hygiene, while milk composition remained within breed-specific norms. Regular veterinary support (14.3%), post-milking teat dipping (34.9%), and hygiene record-keeping (9.6%) were the most underused practices, and farmer education showed the strongest, though non-significant, gradient with SCC (Kruskal–Wallis H = 9.13, *p* = 0.058).

Taken together, these findings define three high-leverage, low-cost priorities for the sector: periodic bulk-tank SCC reporting with farm-level benchmarking against EU thresholds; structured preventive veterinary advisory services integrated into the Common Agricultural Policy advisory framework; and education-sensitive, peer-facilitated training focused on post-milking teat dipping and dry-off management. Embedded within a One Health perspective and supported by companion evidence on antimicrobial-resistant staphylococci and pathogenic E. coli from the same region, such measures represent a cost-effective investment in animal health, farm economic resilience, and dairy food safety. Confirmation in larger, longitudinal, species-stratified studies would consolidate the directional trends observed here and guide their translation into policy across comparable Mediterranean small ruminant production systems.

## Figures and Tables

**Table 1:** Physicochemical and microbiological parameters of bulk-tank raw milk (n = 80 farms). SCC: somatic cell count; TBC: total bacterial count; SD: standard deviation.

Parameter	n	Mean ± SD	Median	Range
pH	80	6.51 ± 0.11	6.51	5.80–6.90
Freezing point depression (°C)	79	−0.597 ± 0.038	−0.600	−0.670 to −0.484
Fat (%)	80	5.82 ± 1.54	5.73	1.19–11.21
Lactose (%)	79	4.82 ± 0.21	4.83	3.77–5.33
Protein (%)	79	5.35 ± 0.76	5.38	3.06–6.44
Solids—non-fat (%)	78	11.07 ± 0.82	11.22	8.33–12.33
Total solids (%)	80	16.83 ± 2.07	17.13	11.77–23.13
SCC (×10^3^ cells/mL)	80	1123 ± 913	883	21–5467
TBC (×10^3^ CFU/mL)	80	496 ± 1133	166	1–8672

**Table 2:** Distribution of farms by SCC-based mastitis classification (n = 80 farms).

SCC Category	Threshold (×10^3^ Cells/mL)	n	%
Healthy (Negative)	≤200	8	10.0
Subclinical (Traces)	201–400	6	7.5
Serious—Stage 1	401–1200	40	50.0
Serious—Stage 2	1201–5000	25	31.2
Serious—Stage 3	>5000	1	1.2
Total	-	80	100.0
Exceeding EU limit (>1500)	-	21	26.2

**Table 3:** Distribution and summary statistics of bulk-tank total bacterial count (TBC) (n = 80 farms). SD: standard deviation.

Parameter	Value
Mean ± SD (×10^3^ CFU/mL)	496 ± 1133
Median (range) (×10^3^ CFU/mL)	166 (1–8672)
Farms ≤ 500 × 10^3^ CFU/mL, n (%)	62 (77.5)
Farms > 500 × 10^3^ CFU/mL, n (%)	18 (22.5)
Farms > 1500 × 10^3^ CFU/mL, n (%)	5 (6.2)

**Table 4:** Spearman rank correlations between SCC and selected milk quality and farm parameters. ***: *p* < 0.001.

Variable 1	Variable 2	n	Spearman ρ	*p*-Value
**SCC**	TBC (CFU/mL)	80	0.549	<0.001 ***
SCC	Fat (%)	79	0.038	0.739
SCC	Protein (%)	78	−0.111	0.328
SCC	Lactose (%)	79	−0.075	0.509
SCC	Total solids (%)	79	0.079	0.489
SCC	pH	80	0.165	0.140
SCC	Flock size	80	0.201	0.081

**Table 5:** Farmer profile and farm demographic characteristics (n = 83).

Characteristic	Category/Statistic	n (%)
Farmer age (years)	Mean ± SD (range)	50.0 ± 11.8 (24–87)
Sex	Male	83 (100%)
Education level	No formal education	3 (3.6%)
	Primary school	27 (32.5%)
	Lower secondary (Gymnasium)	14 (16.9%)
	Upper secondary (Lyceum)	32 (38.2%)
	University or above	7 (9.0%)
Years of experience	Mean ± SD (range)	24.3 ± 12.7 (2–60)
Primary income source	Exclusively livestock	61 (73.5%)
	Mixed (livestock + off-farm)	18 (21.7%)
	Primarily off-farm	4 (4.8%)
Herd size category	Small (<100 animals)	17 (20.5%)
	Medium (100–200 animals)	23 (27.7%)
	Large (>200 animals)	43 (51.8%)

**Table 6:** Animal health management practices reported by farm operators (n = 83).

Health Management Practice	Category/Statistic	n (%)
Milking method	Milking machine (pipeline)	79 (95.1%)
	Hand milking	4 (4.9%)
Veterinary support frequency	Regular (≥quarterly)	12 (14.3%)
	Occasional (only when sick)	37 (44.6%)
	None/ad hoc only	34 (41.0%)
Self-reported mastitis (last 6 months)	Yes	43 (51.8%)
	No	40 (48.2%)
Antibiotic treatment rate (%)	Mean ± SD (range)	6.2 ± 7.0 (0–35%)
Post-milking teat dipping	Yes	29 (34.9%)
Dry-off antibiotic therapy	Yes	18 (21.7%)
Brucellosis vaccination	Yes (mandatory)	83 (100%)
Clostridial vaccination	Yes	37 (44.6%)
Respiratory disease vaccination	Yes	24 (28.9%)
Foot-bathing	Yes	26 (31.3%)
Milking hygiene record-keeping	Yes	8 (9.6%)

**Table 7:** Associations between farmer profile variables and management practices with bulk-tank SCC (n = 80). ns = not significant after Bonferroni correction. Sample sizes for each level of the categorical factors are reported in Table 4 and Table 5 (and, for education, in the Figure 3 caption). Benjamini–Hochberg FDR-adjusted *p*-values did not alter the significance status of any test.

Variable	Test	Statistic	*p*-Value	Direction
Farmer education level	Kruskal–Wallis H	H = 9.13, df = 4	0.058	Higher ed → lower SCC
Post-milking teat dipping	Mann–Whitney U	U = 416.0	0.104	Teat dip → lower SCC
Regular vet support	Mann–Whitney U	U = 168.0	0.149	Reg. vet → lower SCC
Antibiotic treatment rate	Spearman ρ	ρ = −0.197	0.152	Higher rate → lower SCC (ns)
Self-reported mastitis	Mann–Whitney U	U = 486.0	0.218	Reported → higher SCC (ns)
Farmer age	Spearman ρ	ρ = −0.075	0.503	No clear trend
Years of farming experience	Spearman ρ	ρ = −0.062	0.574	No clear trend
Herd size category	Kruskal–Wallis H	H = 1.83, df = 2	0.400	No clear trend
Dry-off therapy	Mann–Whitney U	U = 303.0	0.289	No clear trend
Production system	Kruskal–Wallis H	H = 2.16, df = 3	0.540	No clear trend

## Data Availability

The dataset supporting the conclusions of this article is available from the corresponding author upon reasonable request, subject to applicable data protection regulations.

## Supplementary Materials

The following supporting information can be downloaded at https://www.mdpi.com/article/10.3390/ani16132030/s1, File S1: Sensitivity analyses on the full dataset—residual diagnostics (Shapiro–Wilk; Breusch–Pagan), AIC/BIC-based model selection, internal validation by k-fold cross-validation and bootstrapping, a region random-effect mixed model, an ordinal logistic model of the SCC mastitis-stage categories, and Benjamini–Hochberg FDR control.
