# Supplementary material for: Raw Milk Quality and Subclinical Mastitis Burden in Small Ruminant Farms in Northwestern Greece: A Cross-Sectional Study
Source: Animals (Basel). 2026 Jul 2;16(13):2030. doi: 10.3390/ani16132030 (PMC13359843; doi:10.3390/ani16132030)
Supplement: Supplementary file 1 [file animals-16-02030-s001.zip › animals-4388120-supplementary.pdf]

Supplementary Material S1

Statistical Robustness and Sensitivity Analyses

Manuscript animals-4388120

Note on scope and data. The analyses below assess the robustness of the study's conclusions to a range of statistical considerations. They were computed on the full official project database (n = 81 farms with valid bulk-tank SCC; n = 61 complete cases for the multivariable models). To avoid altering the primary published analysis, these results are reported here as sensitivity/robustness checks and are not intended to replace the figures in the main text. Minor numerical differences relative to the main analysis reflect the inclusion of all available records and the absence of management-practice covariates (see final note). Software: Python (pandas, scipy, statsmodels, scikit-learn).

1. Multiple-testing correction

The ten farm-level bivariate tests were re-evaluated under Holm and Benjamini–Hochberg (BH) false discovery rate (FDR) control. As shown in the main text, no test reached significance under any correction. On the full dataset the bivariate set that could be reconstructed from the unambiguously coded variables gave:

Association (test)	raw p	Holm	BH-FDR
SCC ~ education (Kruskal–Wallis)	0.082	0.495	0.288
SCC ~ TBC (Spearman)	<0.001	<0.001	<0.001
SCC ~ size (Kruskal–Wallis)	0.241	1.000	0.562
SCC ~ farmer age (Spearman)	0.505	1.000	0.884
SCC ~ region (Mann–Whitney)	0.942	1.000	0.976
SCC ~ species (Kruskal–Wallis)	0.976	1.000	0.976
SCC ~ production system (Kruskal–Wallis)	0.967	1.000	0.976

The SCC–TBC association remained highly significant under FDR control; no demographic/structural factor was significant. This confirms that the non-significance of the management/profile associations is not an artefact of an overly conservative Bonferroni correction.

2. Effect size with confidence interval

SCC by education (Kruskal–Wallis): H = 6.69, p = 0.082; effect size $\varepsilon^2 = 0.059$, bootstrap 95% CI [−0.025, 0.270] (2,000 resamples). The interval includes zero on the full dataset, consistent with a small-to-moderate but imprecisely estimated effect; the point estimate and direction (higher education → lower SCC) match the main analysis. Reporting magnitude with its CI, rather than a binary p-value verdict, is the appropriate framing.

3. Regression assumptions and diagnostics

Model: OLS of log10(SCC) on education, farmer age, species, herd-size category and region (n = 61).

Diagnostic	Result
Shapiro–Wilk on residuals	W = 0.886, p < 0.001 → residuals NOT normal
Breusch–Pagan (homoscedasticity)	LM = 3.86, p = 0.695 → homoscedastic (assumption met)
Model fit	$R^2 = 0.032$, adjusted $R^2 = -0.075$

The log10 transformation did not yield normal residuals; variance was, however, homogeneous. The negligible (and negative adjusted) R^2 indicates that the structural/demographic predictors explain essentially none of the variance in SCC — consistent with the manuscript's conclusion that the study is underpowered and that unmeasured factors dominate.

4. Model selection and internal validation

Information criteria favour the most parsimonious specification, and internal validation shows no out-of-sample predictive validity, indicating a substantial over-fitting risk.

Model	AIC	BIC
log10(SCC) ~ education	95.4	99.6
… + age	96.7	103.0
… + species	99.8	110.3
… + species + size + region (full)	103.7	118.5

Internal validation: apparent R^2 = 0.032; 5-fold cross-validated R^2 was strongly negative (unstable across folds owing to the small sample); bootstrap optimism-corrected $R^2 \approx -0.20$ (mean optimism 0.23). The model therefore has no demonstrable predictive validity, and backward elimination by p-value is confirmed to over-fit. The regression is best reported as exploratory/descriptive, not predictive.

5. Accounting for clustering: mixed-effects and ordinal models

Linear mixed-effects model (log10(SCC) ~ education + age + species + size, with a random intercept for region): education coefficient = −0.100 (SE 0.091, $p = 0.273$, 95% CI [−0.279, 0.079]); the region random-effect variance was ≈ 0 (on the boundary), i.e. negligible between-region variance after fixed effects. Accounting for the Ioannina/Preveza imbalance does not alter the (null) education effect, indicating the OLS standard errors were not materially biased by regional clustering in this sample.

Ordinal logistic regression (proportional-odds model of the five SCC mastitis stages on education and age, $n = 61$): education OR = 0.756, 95% CI [0.412, 1.387], $p = 0.366$. Higher education is associated with lower odds of a more severe mastitis stage — the same direction as the main analysis — but the association is not statistically significant. The ordered-outcome specification thus corroborates the descriptive trend without changing the inferential conclusion.

6. Total bacterial count: central tendency

Total bacterial count (full dataset, n = 81): median = 176 × 10^3 CFU/mL, interquartile range = 49–405 × 10^3 CFU/mL (IQR = 356), range 1–22,926 × 10^3 CFU/mL. Given the extreme right-skew (CV > 200%), the median and IQR are the appropriate descriptors; the mean is retained in the main text only for comparability with prior literature.

Overall conclusion

Across every robustness check — FDR control, effect-size estimation with confidence intervals, residual diagnostics, information-criterion selection, internal validation, mixed-effects modelling and an ordinal-outcome specification — the substantive conclusions of the study are unchanged: there are no statistically significant associations between farmer profile/structural factors and bulk-tank SCC, the education effect is directionally consistent but small and imprecise, and the multivariable model is exploratory rather than predictive. The checks therefore strengthen, rather than overturn, the manuscript's interpretation of underpowered, directional trends.
